# Effect of Semen on Vaginal Fluid Cytokines and Secretory Leukocyte Protease Inhibitor

**DOI:** 10.1155/2008/820845

**Published:** 2008-07-03

**Authors:** Kathy J. Agnew, Jan Aura, Norma Nunez, Zandra Lee, Rick Lawler, Carol E. Richardson, Jennifer Culhane, Jane Hitti

**Affiliations:** ^1^Department of Obstetrics and Gynecology, University of Washington, 1959 NE Pacific Street, Box 356460, Seattle, WA 98195, USA; ^2^Department of Obstetrics and Gynecology, Fred Hutchinson Cancer Research Center, 1100 Fairview Avenue North, P.O. Box 19024, Seattle, WA 98109-1024, USA; ^3^Drexel College of Medicine, Drexel University, OB/GYN Control, 245 N 15th Street Philadelphia, PA 19102, USA

## Abstract

The presence of semen in vaginal fluid, as identified by an acid phosphatase spot test, does not influence vaginal proinflammatory cytokine concentrations. *Objective*: determine whether semen, as detected by acid phosphatase, influences vaginal cytokines or secretory leukocyte protease inhibitor concentrations. *Methods*: 138 pregnant women had vaginal fluid collected for Gram stain, acid phosphatase detection by colorimetric assay, and interleukin 1-Beta, interleukin-6, interleukin-8, and secretory leukocyte protease inhibitor measurement by enzyme immunoassay. Results for women with and without acid phosphatase were compared by Mann-Whitney test. *Results*: of 138 subjects, 28 (20%) had acid phosphatase detected; of these, only 19 (68%) reported recent intercourse and 3 (11%) had sperm seen on Gram stain. There were no significant differences in proinflammatory cytokine concentrations; however, secretory leukocyte protease inhibitor concentrations were significantly higher among women with acid phosphatase. *Conclusions*: proinflammatory cytokine measurement does not appear to be affected by the presence of semen, but secretory leukocyte protease inhibitor is significantly higher when semen is present. Detection of semen by acid phosphatase was associated with higher vaginal SLPI concentrations, however, the presence of semen did not appear to influence vaginal proinflammatory cytokine concentrations.

## 1. INTRODUCTION

Bacterial vaginosis (BV) is a common condition causing various symptoms such as vaginal discharge, odor and irritation, and has been associated with increased acquisition of many sexually transmitted diseases [1, 2]. Pregnant women with BV have an increased risk of preterm labor and preterm delivery with the potential for neonatal morbidity and mortality [3]. Early research concerning BV characterized the vaginal microbiological flora using Gram stain and culture [4, 5]. More recent studies have focused on host defense factors and the local immune response that may mediate the relationship between vaginal flora and adverse reproductive and pregnancy outcomes [6, 7]. Proinflammatory cytokines such as interleukin 1-Beta (IL-1*β*), interleukin 6 (IL-6), interleukin 8 (IL-8), and the host defense molecule secretory leukocyte protease inhibitor (SLPI) have been of particular interest [8–10].

Subjects are routinely asked to avoid vaginal intercourse and the use of intravaginal products that might affect test results prior to having specimens collected. However, compliance with these requests is difficult to assess. When vaginal fluid is collected to measure cytokine concentrations, it is important to determine what effect, if any, there may be on the results if semen is present. The objective of this analysis was to determine whether semen present in vaginal fluid alters proinflammatory cytokine or SLPI concentrations. We hypothesized that the presence of semen would increase the concentrations of vaginal proinflammatory cytokines and SLPI.

## 2. MATERIALS AND METHODS

This secondary analysis included data from 138 pregnant women, between 7 and 20 weeks gestation, who participated in a prospective observational cohort study of the effects of BV on pregnancy outcome. Subjects were recruited from the prenatal clinics associated with the University of Washington Medical Center in Seattle, Wash, USA. Participation in the study was limited to those subjects who met the following criteria: singleton pregnancy less than 20 weeks gestation, no prior preterm birth or major medical problems such as chronic hypertension or pre-existing diabetes, and no recent antibiotic use. The study was approved by the University of Washington and the Centers for Disease Control and Prevention Institutional Review Boards and all subjects provided written, informed consent.

The data for the present analysis were taken from study entry visit. We compared subject history, Gram stain for sperm and detection of acid phosphatase as predictors for the presence of semen in vaginal fluid. Acid phosphatase was considered to be the reference as it is an enzyme present in high concentrations in semen, but not found in other secretions such as vaginal fluid, saliva, or mucus [11]. We then compared the concentrations of proinflammatory cytokines and SLPI in samples from women with and without semen detected in vaginal fluid.

Subjects were asked to abstain from vaginal intercourse and the use of intravaginal products for 48 hours prior to their study visit. Subjects completed a structured interview with questions regarding demographics, reproductive history, behavioral habits, and time of last intercourse. A physical exam was conducted including notation of Amsel criteria [12] as well as a vaginal wet mount and Gram stain.

Two Dacron swabs were used to collect vaginal fluid from the posterior vaginal fornix and placed in cryotubes containing 0.9 mL phosphate buffered saline. Swabs were frozen at −80 degrees and stored for later cytokine and SLPI testing. An additional Dacron swab was used to collect vaginal fluid to prepare an air-dried microscope slide which was then Gram stained and read at 100X magnification for the presence of semen and determination of BV score by Nugent criteria [13]. Vaginal fluid from the frozen samples was aliquoted and used to measure proinflammatory cytokine and SLPI concentrations by enzyme immunoassay [14]. For acid phosphatase detection, vaginal fluid was spotted to Whatman no.1 filter paper and then placed in a chemical fume hood and sprayed until wet with the prepared reagent. Development of a purple color within 1 minute was considered a positive test for the presence of acid phosphatase [15, 16]. The reagent was prepared by mixing 10 mL of stock solution A (1 gram Fast Blue B, 20 grams sodium acetate trihydrate, 10 mL glacial acetic acid, 100 mL dH_2_0) and 1.0 mL of stock solution B (0.4 grams sodium alpha naphthyl acid phosphate, 5 mL dH_2_0) in a spray bottle. The prepared reagent has a shelf life of 7 days, while stock solutions A and B are stable for up to six months at 4 degrees.

We used the Chi square test or Fisher’s exact test for categorical variables. The Mann-Whitney test was used for continuous variables. Analyses were stratified by presence or absence of BV on Gram stain, given that proinflammatory cytokines and SLPI may vary with BV status [17, 18].

## 3. RESULTS

Of 138 subjects, 36 (26%) reported vaginal intercourse within the past two days, despite instructions to abstain. 28 (20%) had acid phosphatase detected in their vaginal fluid, and 6 (4%) had sperm seen on Gram stain. Women with detectable acid phosphatase were more likely than those without acid phosphatase to report recent vaginal intercourse (68% versus 15%, *P* < .0001), yet subject history and acid phosphatase detection did not have high concordance. Subjects with and without detectable semen by acid phosphatase were similar with respect to age, race/ethnicity, gestational age, and presence of genital infections (see Table 1).

**Figure 1:**
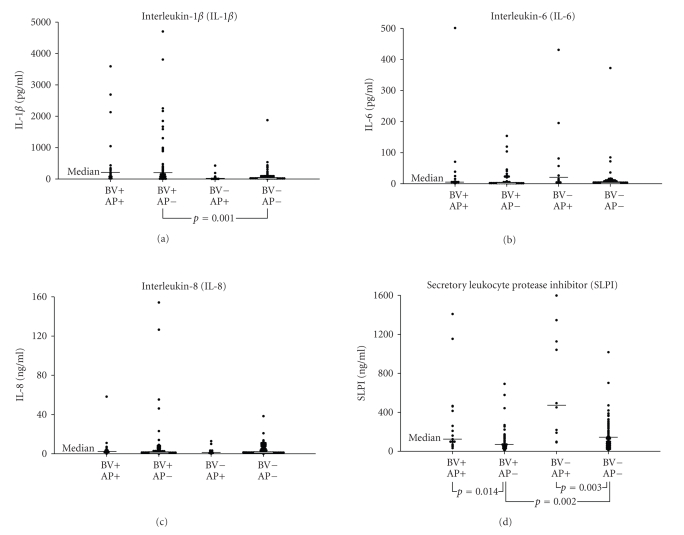
Comparison of vaginal cytokine and secretory leukocyte protease inhibitor concentrations by presence or absence of acid phosphatase and bacterial vaginosis.

The remaining analyses compared vaginal cytokine and SLPI concentrations among women with and without acid phosphatase detected in vaginal fluid. There was no significant difference in IL-1*β*, IL-6, or IL-8 concentrations among women with or without acid phosphatase, when stratified for BV (see Figures 1(a), 1(b), 1(c)). However, SLPI concentrations were significantly higher in the acid phosphatase positive group regardless of BV status (see Figure 1(d)).

There was a significant increase in IL-1*β* and decrease in SLPI among women with BV (see Figures 1(a), 1(d))

## 4. DISCUSSION

In this cohort of pregnant women, detection of semen by acid phosphatase was associated with higher SLPI concentrations in vaginal fluid. These results are not surprising, in that SLPI is a known component of seminal fluid, [19] however, the presence of semen did not appear to influence the vaginal proinflammatory cytokine concentrations. Proinflammatory cytokines can also be detected in semen but are usually not present at high concentrations except in the context of a sexually transmitted infection in the male partner. Increased cytokine levels in semen have been established in HIV infection (IL-1*β*), [20] and genital infections such as *Chlamydia trachomatis* (IL-8) [21] and *Neisseria gonorrhoeae* (IL-6, IL-8) [22].

We did find a high (26%) prevalence of recent vaginal intercourse by history, despite instructions to abstain prior to the study visit. We also found that patient history is not a highly sensitive or specific marker for the detection of acid phosphatase, as only 68% of those with acid phosphatase present reported recent intercourse. Gram stain detection of sperm is limited by the concentration of sperm as well as duration of persistence in the vaginal fluid. In addition, we found that Gram stain is not a useful method to screen for recent vaginal intercourse, with a sensitivity of only 11%. While other tests such as prostate specific antigen (PSA) may be more sensitive, the cost and implementation of equipment and methodology to perform this ELISA test may be prohibitive to many laboratories. It is necessary to note that while samples were immediately frozen for storage after collection and care taken to minimize the number of freeze/thaw cycles when aliquoting for testing, we can not determine if there was any degradation of cytokine concentrations over time or due to the presence of semen. Also our findings may not be generalizable to a nonpregnant population.

Results from this study suggest that the use of acid phosphatase to detect semen in vaginal fluid samples can provide useful information and this testing can be performed using a simple and inexpensive method. Vaginal fluid samples with acid phosphatase should not have SLPI measured, as there is likely a substantial contribution from seminal fluid. However, measurement of proinflammatory cytokines is probably not influenced by the presence of semen. The acid phosphatase detection test may be a useful adjunct for analysis of the effect of seminal fluid on other host defense and immune factors in future studies.

## Figures and Tables

**Table 1:** Subject characteristics by acid phosphatase.

Subject characteristics	Acid phosphatase + *n* = 28	Acid phosphatase − *n* = 110
Age (median)	27	26
Race/Ethnicity
White	11 (42)	43 (39)
African American	1 (4)	22 (20)
Hispanic	8 (31)	15 (14)
Asian	4 (15)	22 (20)
Other/unknown	2 (7)	8 (7)
Gestational age (median weeks)	16	16
Bacterial vaginosis	18 (64)	51 (46)
*Trichomonas vaginalis *	1 (4)	1 (10)
*Chlamydia trachomatis* **	1 (5)	3 (3)
*Neisseria gonorrhoeae*	0 (0)	0 (0)
Self-reported vaginal intercourse	19 (68)	17 (15)
Sperm on Gram stain	3 (11)	3 (3)

* All data are presented as *n* (%) unless otherwise noted. ** *n* = 21 for AP + group, *n* = 91 for AP − group.
